# Supplementary material for: “They recognize me as a doctor”: evaluation of an HIV peer mobilisation training programme to promote oral HIV self-testing and referral for acute HIV infection screening among gay and bisexual men and transgender women in coastal Kenya
Source: medRxiv. 2025 Apr 10:2025.04.08.25324345. Preprint. [Version 1] doi: 10.1101/2025.04.08.25324345 (PMC12036398; doi:10.1101/2025.04.08.25324345)
Supplement: Supplement 1 [file NIHPP2025.04.08.25324345v1-supplement-1.pdf]

medRxiv preprint doi: https://doi.org/10.1101/2025.04.08.25324345; this version posted April 10, 2025. The copyright holder for this preprint (which was not certified by peer review) is the author/funder, who has granted medRxiv a license to display the preprint in perpetuity.
It is made available under a CC-BY 4.0 International license .

Annex 1: Supplementary Table 1. Eligibility criteria of mobilised participants and index participants

**Study population**	**Eligibility criteria**
Mobilised participants	Inclusion criteria:<br>• Age 18 years or older;<br>• Male sex assigned at birth;<br>• Reporting oral or anal sex with men in the previous six months;<br>• HIV negative or of unknown HIV status before doing the self-test;<br>• Willing to provide a blood sample;<br>• Reporting at least one behavioural factor or at least two AEHI symptoms or at least one STI symptom[a]<br>○ Behavioural factors: in the previous seven days: any condomless sex; or in the previous three months: sex with only men, receptive anal sex or group sex;<br>○ AEHI symptoms: in the previous 14 days: fever, diarrhoea, fatigue, body aches, sore throat;<br>○ STI symptoms: in the previous 14 days: genital ulcer, genital discharge or anal discharge<br>Exclusion criteria:<br>• Well-adherent to PrEP
Index participants	Inclusion criteria:<br>• Age 18 years or older;<br>• Male sex assigned at birth;<br>• Reporting oral or anal sex with men in the previous six<br>• months;<br>• Being newly diagnosed with HIV, either after peer<br>• mobilisation, AEHI screening, HPN or at the HTC of one<br>• of the study clinics during the study period (April through<br>• August 2019);<br>• Willing to provide a blood sample

AEHI, acute or early HIV infection; PrEP, pre-exposure prophylaxis; STI, sexually transmitted infection. a. Based on published behavioural PrEP eligibility score (Wahome E et al. An Empiric Risk Score to Guide PrEP Targeting Among MSM in Coastal Kenya. AIDS Behav. 2018;22(Suppl 1):35-44.) and AEHI symptom score (Sanders EJ et al. Targeted screening of at-risk adults for acute HIV-1 infection in sub-Saharan Africa. AIDS. 2015;29 Suppl 3:S221-30.)

medRxiv preprint doi: https://doi.org/10.1101/2025.04.08.25324345; this version posted April 10, 2025. The copyright holder for this preprint (which was not certified by peer review) is the author/funder, who has granted medRxiv a license to display the preprint in perpetuity.
It is made available under a CC-BY 4.0 International license .

Annex 2: Supplementary Table 2. Additional quotes

Quote	Respondent	Gender	Age (years)
**Experience with the training programme**			
I was taught on how to deal with peers, how to talk to them and the roles of mobilizers, so I learnt a lot and now I can associate with people easily.	Mobiliser	Male	31-35
I think they [training sessions] were very helpful because we used to remind each other of things we might have forgotten because some of us were starting to maybe forget their roles and in those meeting, we help each other out.	Mobiliser	Male	31-35
**Mobilisation skills: *Networking***			
Personally, I wasn't able to talk to people one on one, but it has helped me a lot in terms of socialization and courage. Secondly, I have learnt to be discreet and conceal one's secret.	Mobiliser	TGW	26-30
Some approach me and want services… though I am not a doctor they recognize me as a doctor and I help them by taking them to [the clinic].	Mobiliser	Male	21-25
I explained the advantages of knowing your status early and if you're not infected you will be assigned to PrEP and if you are infected you will be assigned to ART, and if you adhere to your medication your virus will be suppressed and there are no disadvantages. Either way, if you test positive or negative its for your own benefit.	Mobiliser	Male	31-35
I get my clients [peers] from my hot spot, it's where we all hustle, and they are people I know so I talk to them, and they accept being my clients. Apart from the hot spot I used to get some from social media in WhatsApp groups.	Mobiliser	TGW	21-25
**Mobilisation skills: *Client Empowerment***			
I met a peer educator at HAPA Kenya [LGBTQ organization], and he told me about KEMRI and oral self-testing. I also showed him the rashes and he empowered me to test for HIV, he gave me a lot of information and support.	Client	Male	26-30
There are some friends of mine who advised me to test. I woke up not feeling so well and decided to come to hospital then I remember what my friends had told me and decided to look for one of them who works here at the clinic.	Client	Male	NA
They were not willing to comply, so I used to tell them about my status and how I was. So they gained confidence from that and they decided to test and accepted to come here.	Mobiliser	TGW	26-30
What I understood from the conversation is that, it mostly touched on one's health as an adult. I could not ignore such information.	Client	Male	26-30

medRxiv preprint doi: https://doi.org/10.1101/2025.04.08.25324345; this version posted April 10, 2025. The copyright holder for this preprint (which was not certified by peer review) is the author/funder, who has granted medRxiv a license to display the preprint in perpetuity.
It is made available under a CC-BY 4.0 International license .

That's when I decided to come to KEMRI for the services, and when I came, I was served well and I confirmed my results to be truly HIV positive. He really helped me know my status.			
I would talk to my peers and tell them that their health is important, and they alone can make a different and take good care of their health. We [as mobilizers] mobilize them and it's their choice if they want to live a healthy life or not so I would encourage them to think about their health first.	Mobiliser	TGW	29
He looked at my condition and advised me to come for testing, but I was not convinced at all. In a period of two weeks he came to me again and told me it was important to come to the hospital and seek help, that's when I gave myself courage and came along with him to this place and was tested for HIV.	Client	Male	NA
If you hide you will not only be hiding yourself but you will be threatening your health which could be easy if you could open up to the mobiliser, because I opened up to the mobiliser, he had the courage to explain to me what is going on, which I felt supported that's why I took the step to seek for treatment through him.	Client	Male	NA
He told me the importance of knowing your HIV status, and if you know your HIV status you'll also get to know your partners HIV status at the same time.	Client	Male	25
**Facilitators and motivations for HIV testing**			
There are some friends of mine who advised me to test. I woke up not feeling so well and decided to come to hospital then I remember what my friends had told me and decided to look for one of them who works here at the clinic.	Client	Male	NA
They [clients] like tell me that maybe they feel pain when they urinate and its then I start asking them questions and try to link the symptoms and inform them on the benefits of having early treatment before they become chronic and that maybe they are STIs.	Mobiliser	TGW	26
The reason why I came to get tested is that, I was not feeling well and there were some changes happening, which to me were not normal that's why I went to get tested for HIV.	Client	Male	26
On listening since I was close to the group I thought no this concerns my health, I joined them. When I listened, the information was good and I decided to join in and do a HIV test which turned positive, and we agreed to come to KEMRI clinic for further tests to confirm this status.	Client	Male	22
**Challenges with mobilisation:** ***Misconceptions regarding OST and AHI***			
When I received the results, I didn't believe them. I didn't trust the OST completely since I knew that if one is tested that blood must be drawn first. That's why I ignored the OST results and asked the peer mobiliser to take me with the others to [the clinic] and he agreed.	Client	Male	22

medRxiv preprint doi: https://doi.org/10.1101/2025.04.08.25324345; this version posted April 10, 2025. The copyright holder for this preprint (which was not certified by peer review) is the author/funder, who has granted medRxiv a license to display the preprint in perpetuity.
It is made available under a CC-BY 4.0 International license .

Some of my clients were used to finger prick so they were really not familiar with OST… they find it confusing but to some it was an easy option because they did not want anything to do with blood… but then you tell them if they want to know more it is best for them to do the RNA test.	Mobiliser	TGW	25
Firstly, you tell a client about AHI and the symptoms and that they are malaria-like, so clients don't see the need to test because you used 'malaria-like' and they just take painkillers and feel a little better and they don't bother about testing, so they are in denial and ignore that it might be AHI.	Mobiliser	TGW	26-30
**Challenges with mobilisation:** ***Logistical and financial issues***			
What I am suggesting is AHI and mobilization targets should be different because the mobilisers might lose focus and find it hard to do his job or even give an inaccurate feedback.	Mobiliser	TGW	21-25
My challenge was finding the right time for my clients [peers] because most of them are working class and they are only available during the weekends and in the evening, and they sometimes suggest that the doctors personally take those services to them.	Mobiliser	TGW	21-25
**Challenges with mobilisation:** ***Stigma and security concerns***			
Some of my clients were not ready to come to the centre because if they are seen accompanied by you they are afraid of being judged and questioned why they are talking to a gay… so I used to walk ahead and they would follow me.	Mobiliser	Male	31-35
In terms of harassment, Kilifi station is not pre because most people around there are homophobic and the moment you approach someone and they say you are gay you will get beaten up or even burned alive so you just have to hold back because if you even dare to be persistent and try to persuade them, they might cause you harm.	Mobiliser	TGW	21-25
My experience with trans[gender women] is that they mostly stay indoors during the day… and when people come across a trans person they will question their gender… and they might get beaten up and so… they are afraid of what will happen to them.	Mobiliser	TGW	21-25
**Recommendations for mobilisation:** ***Continued involvement of mobilisers***			
We should not be forgotten because we already have peers, and some are already used to us… we will have a hard time cutting off communication with clients and they will lose morale in their regular checkups ad we will not be able to make follow ups because of money.	Mobiliser	Male	18-20
Mobilizers should be involved in new projects and be given priority in such matters, because it is us who have peers and recruit them and even already have established a strong connection with some, so we should be involved more on matters concerning new projects and get more training to make us more efficient with our work.	Mobiliser	TGW	21-25

medRxiv preprint doi: https://doi.org/10.1101/2025.04.08.25324345; this version posted April 10, 2025. The copyright holder for this preprint (which was not certified by peer review) is the author/funder, who has granted medRxiv a license to display the preprint in perpetuity.
It is made available under a CC-BY 4.0 International license .

What I think is we need more funds to still make follow ups on clients who did not qualify for the study so as to make them feel appreciated and maintain a close connection with them.	Mobiliser	Male	18-20
What I suggest is as this project is coming to an end we need a new project because these peers are already used to us because we some need to reach to more people who are unaware too.	Mobiliser	TGW	21-25
**Recommendations for mobilisation:** ***Additional training and dissemination***			
Going to the health facilities to get to know their HIV status is hard. I'm suggesting that in forums and gatherings we should be told more about HIV, the importance of frequent testing and dangers of not knowing your status in time. If people get to know more about the self-testing kits this will help people feel the ease of getting to know their HIV status and giving them the privacy they need.	Client	Male	26-30
Personally, I think we should be involved in your activities more often and it's not that whenever there is a project is when you involve us. Secondly, we need you to listen to us like the involvement in your projects, in trainings there is the questionnaire and hear out our opinions so we at least feel like we are recognized and it will give us morale.	Mobiliser	TGW	21-25
What I was suggesting is if we could get access to a video clip that shows how the machine operates so we can share the video to clients and they will have a better picture of what the RNA machine does.	Mobiliser	Male	31-35
In my opinion I think RNA test can really encourage people to test, because most of us don't like to test regularly just to confirm if we are actually negative, so this RNA test might boost testing uptake.	Client	Male	26-30
**Recommendations for mobilisation:** ***Tailored outreach to other key and vulnerable populations***			
What I can add on that is trans people are really something we forget to look for, so it is up to us as peer mobilizers to probe for the issues and educate them and have them over the center for their own benefits.	Mobiliser	Male	31-35
I was suggesting for road shows or campaign and so rural people can get to know about it.	Mobiliser	Male	31-35
A very low percent of trans come out in issues regarding HIV services, but if you tell them directly it is hard for them to show up. Mostly when you offer psychosocial support or such it will attract trans people and in the process of them coming, you engage them at HIV activities.	Mobiliser	Male	21-25
